# 5-Lipooxygenase Derivatives as Serum Biomarkers of a Successful Dietary Intervention in Patients with NonAlcoholic Fatty Liver Disease

**DOI:** 10.3390/medicina56020058

**Published:** 2020-02-03

**Authors:** Marcin Banaszczak, Dominika Maciejewska, Arleta Drozd, Karina Ryterska, Dominika Jamioł Milc, Joanna Raszeja-Wyszomirska, Ewa Wunsch, Pedro González-Muniesa, Ewa Stachowska

**Affiliations:** 1 Department of Human Nutrition and Metabolomics, Pomeranian Medical University, 71-460 Szczecin, Poland; marcin.banaszczak@gmail.com (M.B.); dmaciejewska.pum@gmail.com (D.M.); arleta.drozd@gmail.com (A.D.); superkarina@wp.pl (K.R.); dominikajamiol@interia.pl (D.J.M.); 2 Liver and Internal Medicine Unit, Department of General. Transplant and Liver Surgery of the Medical University of Warsaw, 02-097 Warsaw, Poland; joanna.raszeja@wum.edu.pl; 3 Translational Medicine Group, Pomeranian Medical University, 70-111 Szczecin, Poland; ewa.wunsch@gmail.com; 4 Department of Nutrition, Food Science and Physiology, School of Pharmacy and Nutrition, University of Navarra, 31008 Pamplona, Spain; pgonmun@unav.es; 5 Centre for Nutrition Research, School of Pharmacy and Nutrition, University of Navarra, 31008 Pamplona, Spain; 6 CIBERobn Physiopathology of Obesity and Nutrition, Centre of Biomedical Research Network, ISCIII, 28029 Madrid, Spain; 7 IDISNA, Navarra’s Health Research Institute, 31008 Pamplona, Spain

**Keywords:** derivatives, nonalcoholic fatty liver, oxylipids, steatosis

## Abstract

*Background:* It was previously shown that a bodyweight reduction among patients with nonalcoholic fatty liver (NAFLD) was connected to the lower concentration of arachidonic and linoleic acid derivatives in their blood. We hypothesized that the concentration of these lipids was correlated with the extent of their body mass reduction and, thus, liver steatosis. *Methods:* We analyzed 68 individuals who completed the dietary intervention. Patients were divided into two groups depending on their body mass reduction (more or less than 7%). Before and after the dietary intervention, all patients had the following measurements recorded: body mass, waist circumference, stage of steatosis, fatty liver index, liver enzymes, lipid parameters, insulin and glucose. Concentrations of lipoxins A4 (LTX A4), hydroxyeicosatetraenoic fatty acids (5(S)-HETE, 12(S)-HETE and 16(S)-HETE), hydroxyoctadecaenoic acids (9(S)-HODE and 13(S)-HODE) and 5-oxo-eicosatetraenoic acid (5-oxo-ETE) were measured in serum samples collected before and after the dietetic intervention using high-performance liquid chromatography (HPLC). *Results:* Patients who reduced their body mass by more than 7% revealed a significant improvement in their steatosis stage, waist circumference, fatty liver index, triglycerides and cholesterol. *Conclusion*: A reduction in body mass by more than 7% but not by less than 7% revealed a significant improvement in steatosis stage; waist circumference; fatty liver index; and levels of triglycerides, cholesterol, 5-oxo-ETE and LTXA-4.

## 1. Introduction

Nonalcoholic fatty liver disease (NAFLD) is becoming the most common liver disease worldwide [1,2,3,4]. The manifestation of the disease varies from a simple fatty liver to inflammatory nonalcoholic steatohepatitis (NASH) [4,5], leading to cirrhosis [6,7]. Insulin resistance and obesity are principal risk factors for the development of NAFLD. Insulin resistance is associated with the increased release of polyunsaturated free fatty acids (PUFAs) from phospholipid membranes [8]. PUFAs may be used for hepatic re-esterification to triacylglycerols; however, in the setting of inflammation, they are presumed to be involved in the synthesis of a variety of lipid derivatives—oxylipids [8,9,10]. Oxylipids are bioactive particles that play an essential role in normal cell physiology and during pathological processes, where they are responsible for the induction and regression of inflammation [11]. Lipid derivatives arise from PUFA conversions, mainly arachidonic acid (AA) and linoleic acid (LA), as shown in Supplementary Figure S1. AA and LA are utilized by three families of enzymes: (1) lipoxygenases (5-lipoxygenase (5-LOX), 12-lipoxygenase (12-LOX) and 15-lipoxygenase (15-LOX)) that produce leukotrienes, hydroxyeicosatetraenoic acids (HETE) and hydroxyoctadecaenoic acids (HODE); (2) cyclooxygenases (cyclooxygenase-1; COX-1) and cyclooxygenase-2 (COX-2)) that produce prostaglandins and thromboxanes; and (3) monoxygenases that produce epoxies and HETE [12]. During oxidative stress, enantiomers of oxylipids (e.g., 11(S)-HETE) are endogenously synthesized by nonenzymatic pathways [11]. HODE enantiomers (9(S)-HODE and 13(S)-HODE) can be considered markers of oxidative stress; both of them are produced from LA by hepatic 5-LOX and 15-LOX [8]. HETE content (in different tissues) increases as a result of the catabolism of AA by lipoxygenases: 5-LOX, 12-LOX and 15-LOX [13]. The accumulation of 5(S)- HETE or 15(S)-HETE in the liver may be related to the progression of NASH [8]. The fatty acid 16(S)-HETE is produced through cytochrome P450 [12]. Other AA derivatives such as lipoxin A4 and 5-oxo-ETE are formed by 5-LOX [12,13,14]. Oxoeicosatetraenoic acids (5-oxo-ETE) are formed by 5-hydroxyeicosanoid dehydrogenase (5-HEDH), and can also be generated directly through the actions of cytochrome P450 isozymes. However, the synthesis of 5-oxo-ETE increases in conditions that favor oxidative stress [12], as shown in Table 1.

As shown in a recent study [11], a significant linear relationship exists between a high body mass index (BMI), visceral adipose tissue and proinflammatory 5 and 11(S)-HETE [11]. Obese individuals (i.e., with a BMI > 25 kg/m^2^) are characterized by a higher 5(S)-HETE and 11(S)-HETE concentration than those with a lower BMI [11]. Recently published studies [15,16] describe the changes in LA and AA derivatives in patients with NAFLD in correlation with the progression of the disease [15,16]. For example, Selezneva et al. [17] demonstrated that the rate of production of eicosatetraenoic acid metabolites (5(S)-HETE, 15(S)-HETE and 11(S)-HETE) was different in NASH, alcoholic steatohepatitis and overweight patients. However, there are only a few reports on the changes in HETE and HODE concentrations in patients undergoing weight reduction or diet modification [15,18]. 

Among NAFLD individuals, the reduction of initial bodyweight is important to achieve histological liver improvement: a decrease of 7% ameliorates inflammatory liver disease (NASH) [4]. We previously showed that weight reduction was connected to the concentration of arachidonic acid and linoleic acid derivatives (oxylipids) in blood [15]. In the present study, we hypothesized that the concentration of these lipids was correlated with the extent of body mass reduction (below or above 7% initial body mass) and thus the extent of liver steatosis.

## 2. Materials and Methods

### 2.1. Patients

This study is a secondary analysis study (in the post-intervention period) based on results and biological material collected in the study described in detail in a previous report [26]. From the group of patients selected based on their weight loss (below and above 7% body mass), we filtered out patients’ anthropometric and medical data and frozen plasma samples. The study, as described in [26], was a six-month single-center study conducted at the Pomeranian Medical University in Szczecin, Poland from October 2010 to October 2013. All the patients who consented to participate in the study were put on a six-month diet, and they were scheduled to four visits, as described in detail previously [26]. In this study, we presented only the data from timepoint 0 (at the beginning of the diet) and timepoint 1 (six months after the diet).

The study protocol was approved by the Ethics Committee of the Pomeranian Medical University (Szczecin, Poland, 25 01 2010 KB-0012/09/10), and it conforms to the ethical guidelines of the 1975 Declaration of Helsinki. The volunteers provided written, informed consent before the study. One-hundred and ten individuals who completed all the scheduled visits during the six months of the study were invited to ultrasonography (USG) measurements.

Hepatic steatosis was assessed by a trained physician according to the Hamaguchi score [27] using a high-resolution B-mode abdominal ultrasound scanner (Acuson X300, Siemens, San Jose, CA, USA). A Hamaguchi score ≥ 2.0 was set as the diagnostic criterion for NAFLD. From 94 individuals that reduced their initial body mass over 7%, 52 patients were randomly selected to group I using the Research Randomizer application [28], whereas 16 individuals with a body mass reduction below 7% were included in group II. 

Patients from group I and group II did not reveal significant differences in anthropometric parameters, biochemical parameters and liver steatosis at the beginning of the study (U Mann Whitney Test), except for total cholesterol that differed significantly between the groups (205.75 ± 55.18 for group I vs. 181.75 ± 30.38 mg/dlL for group II; *p* < 0.01).

### 2.2. Anthropometric Measurements, Body Mass Reduction

The analysis of anthropometric measurements (body mass, height) was performed at the Department of Human Nutrition and Metabolomics. Body mass and height were determined using digital scales with a height-measuring tool and medical scales with a stadiometer (Radwag, Poland). BMI was calculated on the basis of these measurements (BMI = bodyweight/height in meters squared; kg/m^2^). Waist circumference was measured using a medical tape measure (midway between the bottom edge of the ribs and the iliac crest).

### 2.3. The Fatty Liver Index ( FLI) 

The Fatty Liver Index (FLI): (FLI = (e0.953*loge (triglycerides) + 0.139*BMI + 0.718*loge (ggt) + 0.053*waist circumference−15.745)/(1 + e0.953*loge (triglycerides) + 0.139*BMI + 0.718*loge (ggt) + 0.053*waist circumference−15.745) × 100) helps to assess the NAFLD patient’s steatosis status [29]. FLI may be useful for select subjects who require intensified lifestyle counseling or for other purposes (e.g., epidemiologic study). In the global population, an FLI ≥ 60 rules in hepatic steatosis as detected by ultrasonography [27].

### 2.4. Dietary Intervention

The diet was calculated individually based on the daily caloric needs of the patient. Patients with a BMI that identified them as overweight or obese received a diet that was reduced by 500 kcal per day (reduced from the total daily energy expenditure (TDEE)). Every patient received a weekly menu plan containing detailed recommendations regarding mealtimes, the content of meals and the portion size, as described previously [26]. A nutritionist validated the dietary records (compliant with the guidelines) according to a corresponding food table and a nutrient database with a 72 h food diary (including two working days and one day free of work) at all checkpoints. 

Regarding the content of the diet: energy was individually calculated; the fat content was 20–30% (as total calories percentage), recommended sources—vegetable fats with a predominance of rapeseed oil and olive oil. Butter and margarine were allowed, while hard, animal fats such as lard were excluded. The carbohydrate content was 55–65% (as total calories percentage), recommended with a low and medium glycemic index; these included whole wheat bread, whole wheat pasta, cereal and brown rice. Sweets were excluded from the diet; the protein content was 15% (as total calories percentage), recommended: poultry, fish (oily fish three times a week), fermented dairy (two times a day), and eggs (four to five times a week). A fiber content of 30–35 g/day was recommended: vegetables and fruit, including three portions of vegetables, two portions of fruit, and whole wheat products. The amount of fluid intake was calculated to be 35 mL/kg of actual bodyweight [26].

### 2.5. Fatty Acid Derivatives Measurement

After overnight fasting, venous blood samples were collected into tubes containing anticoagulant (Ethylenediaminetetraacetic acid (EDTA)). Blood samples were centrifuged at 3500 rpm for 10 min at 4 °C within 2 h of collection. Standard blood biochemical analyses were carried out at the University Hospital Laboratory. The separation of the fatty acid derivatives was performed using Chromatography Infinity 1260 (Agilent Technologies, Santa Clara, CA, USA). The column that we used was Hypersil C18 100 × 4.6 mm BDS column (Thermo Scientific, Waltham, MA, USA, 02451) with reversed phase. The detailed description of the sample preparation and analysis was presented in detail in our previous publication [15]. 

### 2.6. Statistical Analysis

StatViev software version 5.0 (SAS Institute Inc., Cary, NC, USA) was used for the statistical analysis, and all the results were expressed as mean ± standard deviation or median and interquartile range (IQR). As the distribution in most cases deviated from normal (Shapiro–Wilk test), nonparametric tests were used: the Wilcoxon test was performed to analyze the dynamic changes of the fatty acid derivatives between time 0 (t0: the start of the study) vs. time 1 (t1: after six months of the dietary intervention). The U Mann–Whitney test was used for comparisons between the groups, and Spearman’s rank correlation coefficient was used for correlations between parameters. The value *p* < 0.05 was considered significant.

## 3. Results

### 3.1. Changes in the Anthropometric and Biochemical Parameters in Both Groups 

Table 2 shows the changes in anthropometric and biochemical parameters after six months (t0–t1) of the dietary intervention. We observed significant differences between the groups for body mass index reduction, waist circumference, serum AST, triglycerides and cholesterol. In group I, the reduction of body mass was 7.4%, whereas in group II it was 4.7%. Group I significantly reduced liver steatosis, BMI, waist circumference, serum triglycerides and FLI compared to group II.

### 3.2. Changes in the Fatty Acid Derivatives

Patients who reduced their body by mass more than 7% showed a significant decrease of 5-LOX derivatives—LTX A4 and 5-oxo-ETE, as shown in Table 3.

### 3.3. The Concentration of the Lipid Derivatives Correlated With the Parameters of Liver Steatosis

Liver steatosis, ALT and HOMA-IR significantly correlated only with two fatty acids derivatives—5-HETE and 5-oxo-ETE, as shown in Table 4. 

## 4. Discussion

Modification of the patients’ lifestyle with the aim of body mass reduction, i.e., dietary modification and physical activity, is the basis of the actual therapeutic recommendations for NAFLD [3,29,30,31]. The loss of ≥ 7% of the initial body mass significantly improves liver functions [31]. A meta-analysis consisting of 78 studies (38 NASH studies and 40 NAFLD studies, including liver biopsy studies) showed that a 5% reduction in baseline bodyweight contributed to a reduction in histological liver steatosis but without affecting fibrosis. The loss of ≥ 7% of baseline bodyweight significantly improved the histological structure of the liver [30]. We achieved similar results in this study, as in group I, where the reduction of the initial body mass was larger than 7% and ultrasonography revealed a reduction of liver steatosis (1.44 ± 0.64; 1.44 is the change in the Hamaguchi score). Group II showed a lower decrease in their initial body mass less than 7%; hepatic steatosis, as assessed by liver ultrasonography, did not improve after six months. There is still a lack of knowledge regarding the role of oxylipins in NAFLD. Feldstein et al. [10] showed that higher concentrations of 9(S)-HODE, 13(S)-HODE, 9-oxo-ODE and 13-oxo-ODE were detectable in the blood of patients with simple steatosis than in healthy individuals. NASH patients had significantly higher concentrations of 5(S)-HETE and 15(S)-HETE than patients with NAFLD [8]. Puri et al. described 5(S)-HETE and 15(S)-HETE as “lipid markers of NASH” [32]. Maciejewska et al. [15] demonstrated the negative role of some derivatives (especially 5(S)-HETE) in the progression of fatty liver in NAFLD, while Raszeja-Wyszomirska et al. [33] did the same (especially 5, 12 and 15(S)-HETE; 9 and 13(S)-HODE) in alcoholic liver disease.

Are oxylipids suitable indicators of successful body mass reduction? According to our results, it may be possible. For example, it was noted that the concentrations of 9(S)-HODE and 13(S)-HODE in the blood samples were higher among obese individuals and decreased after a successful diet [34]. Additionally, 5(S)-HETE, 12(S)-HETE and 15(S)-HETE concentrations increased proportionally to body mass [11,18,35,36]. In obese patients with T2D and in an animal model, 12(S)-HETE was described as an indicator of inflammatory processes in white adipose tissue [37,38]. 

In this study (in the post-intervention period/study), the oxylipins that were significantly different between both groups were leukotrienes A4 (LTX A4) and oxoeicosatetraenoic acids (5-oxo-ETE). Both compounds are metabolites of proinflammatory 5 Lipoxygenase (5-LOX) and are produced from 5(S)-HETE. Leukotrienes A4 act as anti-inflammatory mediators by inhibiting both neutrophil and eosinophil transmigration into infection sites and by promoting the noninflammatory infiltration of monocytes [12]. The increase in the concentration of 5-oxo-ETE (in patients who reduce their body mass less than 7%) acts as a chemoattractant and eosinophil activator [39] and can be considered as a negative phenomenon because the OXE receptor for this derivative shows a strong expression on the surface of hepatocytes, making them susceptible to inflammation that leads to NAFLD [39].

These results support earlier observations from our group, which demonstrated the potential role of 5(S)-LOX during the early stages and progression of NAFLD [15]. The study carried out on a group of 24 people who suffered from first- and second-stage liver steatosis showed that during the early stages of NAFLD, 5-lipoxygenase played the main role in enzymatic transformations responsible for the further development of the illness. Moreover, 5-lipoxygenase expression was increased in the adipose tissue of obese and NAFLD patients [40]. Reduced levels of 5-LOX derivatives could be associated with body mass reduction and liver steatosis. Our study revealed that oxylipins (5-LOX products) were associated the most with liver steatosis. It seems that the derivatives of 5-LOX may serve as markers of progression for the illness.

In summary, we are the first to postulate that reductions of LTX A4 and 5-oxo-ETE in the blood represent oxylipin serum biomarkers of a successful reduction of body mass that lead to the reduction of liver steatosis in NAFLD patients. 

## 5. Conclusions

The reduction of LTX A4 and 5-oxo-ETE serum levels is associated with successful weight reduction and reduced liver steatosis in NAFLD patients.

## Figures and Tables

**Table 1:** The physiological functions of selected fatty acid derivatives.

Product	Function in the Organism	Research Model	Source
A4 Lipoxins	It affects the reduction of tumor necrosis factor (TNF)-α and Interleukin (IL)-6 in plasma. Decreases liver cell apoptosis.	Mice	[19]
16(S)-HETE	The inhibition of the adhesion and aggregation of neutrophils in the intestines.	Mice, human	[20]
9 13(S)-HODE	A strong correlation with liver histopathology (inflammation, fibrosis and steatosis). An activator of Low density lipoprotein (LDL) oxidation. It affects the increase in the secretion of cholesterol from macrophages.	Mice, human cell line	[10] [21]
5(S)-HETE	The sensitisation of hepatocytes towards apoptosis induced by TNF-α 5. A prominent role in the inflammatory process. Activation of hepatic stellate cells and Nonalcoholic fatty liver disease (NAFLD) progression.	Human cell line, murine	[22]
12(S)-HETE	The stimulation of the expression of Nicotinamide adenine dinucleotide phosphate (NADPH) oxidase on pancreatic islets. The induction of inflammation via the stimulation of protein kinase C.	Cell line Rats	[23] [24]
15(S)-HETE	An inflammation marker.	Rats	[24]
5-oxo ETE	Strong eosinophils’ activator acting as a chemoattractant	Humans	[25]

**Table 2:** The anthropometric and biochemical parameters in both groups of patients after six months (shown as differences). All the values are provided as mean ± standard deviation. The significance of differences was calculated by the U Mann–Whitney test. The level of significance was set as *p* ≤ 0.05.

Δ (t0-t1 )	Group I *n* = 52	Group II *n* = 16	*p*
Δ Body mass index (BMI) (kg/m^2^)	−2.30 ± 1.54	−1.38 ± 1.42	0.04
Δ Waist circumference (cm)	−8.28 ± 13.5	−3.09 ± 5.43	0.03
Δ Stage of steatosis(Hamaguchi score)	−1.44 ± 0.64	0.00	<0.0001
Δ Fatty Liver Index (FLI)	−12.38 ± 13.83	−6 ± 8.48	0.035
Δ AST (U/L)	−4.11± 11.69	1.87 ± 13.54	0.01
Δ ALT (U/L)	−10.65 ± 29.23	−19.12 ± 32.54	0.70
Δ GGTP (U/L)	−16.94 ± 51.16	13.62 ± 81.78	0.25
Δ Triglycerides (mg/dL)	−20.19 ± 93.36	45.37 ± 80.66	0.001
Δ Cholesterol (mg/dL)	−9.46 ± 33.49	21.00 ± 31.57	0.004
Δ High-density lipoproteins (HDL) (mg/dL)	2.65 ± 7.55	2.56 ± 5.27	0.94
Δ Low-density lipoproteins (mg/dL)	−3.19± 43.50	11.06 ± 29.05	0.15
Δ Glucose (mg/dL)	−0.15 ± 12.35	6.06 ± 17.29	0.21
Δ Insulin (U/mL)	−3.77 ± 10.47	−4.42 ± 7.46	0.70

AST: Aspartate transaminase; ALT: Alanine aminotransferase; GGTP: Gamma-glutamyltransferase.

**Table 3:** The analysis of the changes in concentration of the linoleic acid (LA) and arachidonic acid (AA) derivatives between both groups after the six months dietary concentration (U Mann–Whitney test). The level of significance was set as *p* ≤ 0.05.

Eicosanoids μg/mL	Group I, *n* = 52 Δ t0–t1 Median (IQR)	Group II, *n* = 16 Δ t0–t1 Median (IQR )	*p*
LTX A4	−0.09 (1.17)	0.52 (1.02)	0.02
16(S)-HETE	−0.03 (0.35)	0.05 (0.46)	0.45
13(S)-HODE	−0.56 (3.17)	−0.05 (4.35)	0.43
9(S)-HODE	−1.00 (3.72)	−0.16 (5.40)	0.45
15(S)-HETE	−0.93 (7.09)	0.58 (11.28)	0.96
12(S)-HETE	1.52 (10.38)	2.16 (10.86)	0.83
5-oxo–ETE	−0.01 (1.24)	0.73 (1.80)	0.01

**Table 4:** The relation between lipids derivatives concentration and the correlating steatosis parameters in both groups were calculated by Spearman’s correlation coefficient. The level of significance was set as *p* ≤ 0.05.

NAFLD Parameters	Eicosanoids	Rho	*p*
Stage of liver steatosis	5-S HETE	0.28	*p* < 0.05
	5-oxo-ETE	0.29	*p* < 0.05
ALT	5-S-HETE	0.29	*p* < 0.05
	5-oxo-ETE	0.32	*p* < 0.05
HOMA-IR	5-S HETE	0.30	*p* < 0.05
Fatty Liver Index	-	-	-

ALT: Alanine aminotransferase; HOMA-IR: Homeostatic Model Assessment for Insulin Resistance; NAFLD: Nonalcoholic fatty liver.

## Supplementary Materials

The supplementary materials are available online at https://www.mdpi.com/1010-660X/56/2/58/s1. The datasets used and/or analyzed during the current study are available from the corresponding author upon a reasonable request.
