# Supplementary figures and images for: 5-Lipooxygenase Derivatives as Serum Biomarkers of a Successful Dietary Intervention in Patients with NonAlcoholic Fatty Liver Disease
Source: Medicina (Kaunas). 2020 Feb 3;56(2):58. doi: 10.3390/medicina56020058 (PMC7073509; doi:10.3390/medicina56020058)

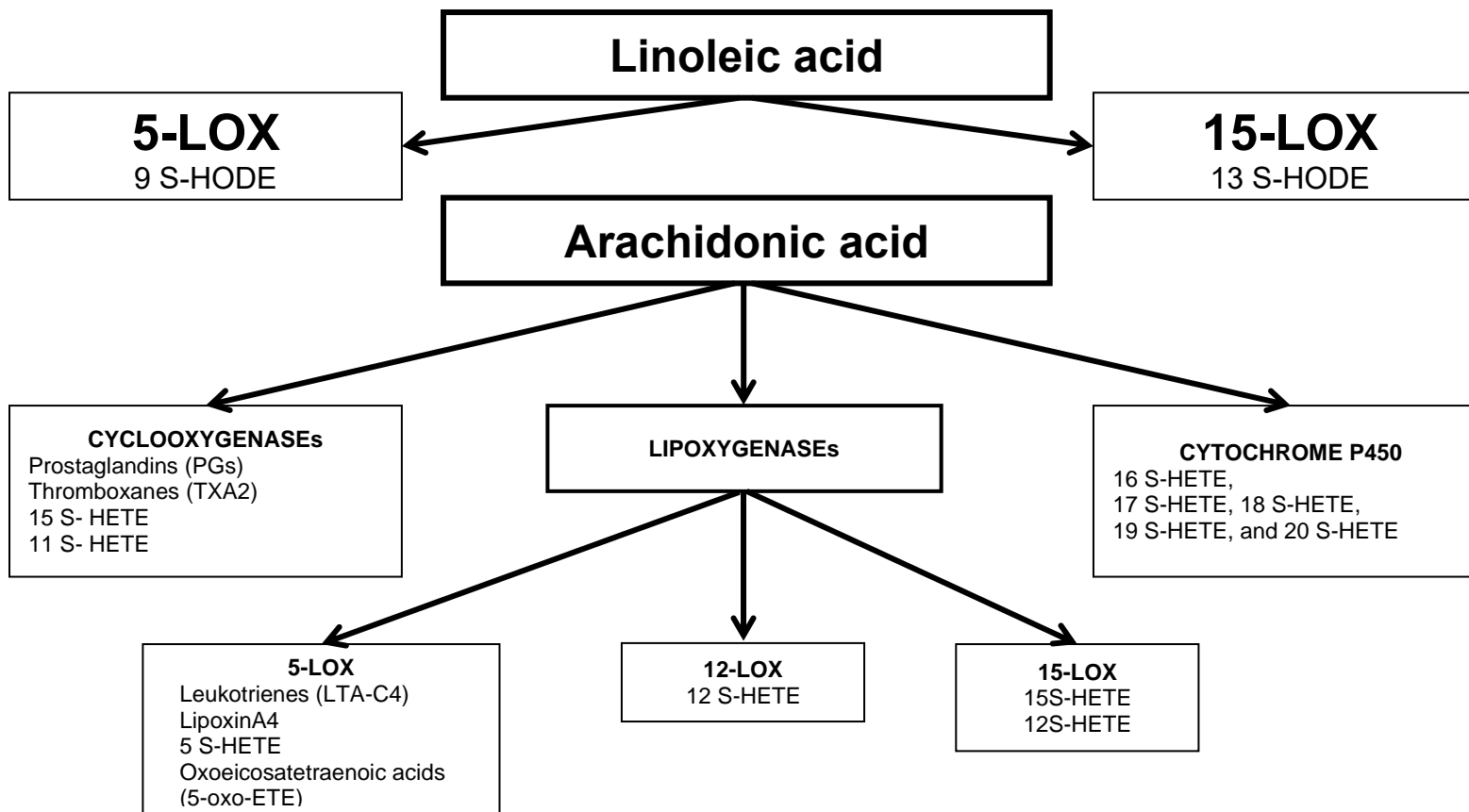

Supplement: Supplementary file 1 [file medicina-56-00058-s001.pdf]
